# Hospital and Institutional News

**Published:** 1918-01-12

**Authors:** 


### HOSPITAL AND INSTITUTIONAL NEWS.

### A NATIVE GENERAL HOSPITAL.

This hospital is situated somewhere in France, and has been organised by Major Frood, a resident of Johannesburg since 1888. He has had a unique experience with native races of all kinds, and is probably one of the ablest managers of these patients in the Service. He has 300 beds available, of which some 180 were in occupation when we had the pleasure of visiting the hospital, and everything was in good order, the hygienic conditions throughout being excellent. Amongst the patients are usually to be found representatives of all African races, in addition to Chinese, Egyptians, and others. An interesting fact which the working of this hospital has established is, that, the "boys" exhibit commendable thrift and save their money, being well content with their rations. This praiseworthy habit on the part of the "boys" has rendered it useless to maintain a canteen, for which, in fact, the patients have no use. The hospital is pleasantly situated on the side of a hill. Everything was in exemplary order, and the discipline throughout of the highest, due, no doubt, to the great experience and capacity of Major Frood, the O.C., and his system of judiciously mixing the patients of various races, which minimises the risk of trouble arising, and secures the best management easily and without difficulty. The patients display great readiness in modelling in clay animals of many types, some of the work being most interesting and characteristic.

### REOPENING OF THE SCIENCE MUSEUM.

The Board of Education announce that the Science Museum, South Kensington, is to be reopened to the public from January 1. The Museum has been closed to the public for nearly two years; though it has, however, been open for students. As compared with 1914 conditions, the extent and the hours of opening for 1918 are somewhat reduced, but the greater part of the Museum will be open free on every weekday from 10 a.m. to 5 p.m., and on Sundays from 2.30 p.m. to 5 p.m. The collections contain many unique objects of great interest as representing discoveries, inventions, and appliances that have been of first-rate importance in the advancement of science and of industry. Such objects as Watt's engines, early locomotives, steamships, flying machines, reaping machines, and textile machinery are records of British contributions to the progress of the world, and it is gratifying that these can again be made available for inspection by visitors to London from all parts of the United Kingdom, and from distant parts of the Empire.

### SOME IMPORTANT BEQUESTS.

A large part of the £90,000 left by Mrs. Susan Paddock, of the Hyde Park Hotel, Knightsbridge, a daughter of Dr. Duncan Matheson, of Manchester, is devoted to charity. £10,000 is bequeathed to St. Mary's Hospital for Women and Children, Manchester, for the endowment of a ward in memory of the testator's father, £1,500 to the Manchester Royal Infirmary and the Manchester Children's Hospital, in both cases for the endowment of beds. Sums of £1,000 each are left to Henshaw's Blind Asylum and the Manchester and Salford Blind Aid Society. Mrs. Paddock made exact arrangements for her burial, and expressed a desire for a choral service and for large quantities of flowers to be used as wreaths and for decoration; the text "I will restore to you the years that the locust hath eaten" is to be inscribed on her tombstone. Mrs. Martineau, of Letchworth, has also left important bequests to hospitals and other charities, including £3,000 to the London Homœopathic Hospital, £500 each to the London Temperance Hospital, the Women's Hospital, Euston Road, and the Cottage Hospital, Letchworth; and £200 each to the Charity Organisation Society, the Anti-Vivisection Hospital, Battersea, the Hahnemann Convalescent Home, Bournemouth, and the Society for the Employment of Epileptics. Nursing Associations are not overlooked, and £50 each was left in the will to the Suffolk Nursing Association and the Paddington and Marylebone Nursing Association.

### OFFICERS AT BOOTHAM PARK.

Dr. Jeffrey, the medical superintendent of Bootham Park, York, who has been temporarily released for military service, and is now at Craiglockhart War Hospital, Edinburgh, has completed five useful years of work at this mental institution. His temporary successor, Dr. Richardson, came from Ticehurst, the large private asylum, before which he had been medical superintendent of the Isle of Man Asylum. Bootham Park has been asked to receive ten cases of mental breakdown among naval and military officers. This request from the Minister of Pensions has been complied with, though mental institutions, like others, suffer from a shortage of staff.

### THE RETENTION OF INDISPENSABLE MEN.

The question as to whether it is better in the national interests to retain in civil life young men who, by their training and industry, have rendered themselves practically indispensable, was raised at Lambeth Tribunal, when Mr. J. L. Goldspink, clerk to the Lambeth Board of Guardians, applied for the exemption of Mr. P. W. Sugden, assistant in the pathological laboratory. He was unmarried, twenty-two years of age, and had originally been medically rejected, but on being called up for another examination was classified as fit for garrison duty at home. He was trained at Guy's Hospital, where he served an apprenticeship of four years, and he came direst from there to the Guardians eighteen months ago. Mr. Goldspink said he was engaged in most important work in the laboratory, which previously had been done by a qualified medical man, and should he go they would have to engage another medical man at a greatly enhanced salary to take his place. This work had increased 68 per cent., the specimens for examination last year amounting to several thousands, and the numbers had since rapidly increased. The Chairman said he did not believe that anybody of the age of this man was doing indispensable work, but Mr. Goldspink pointed out that his training and experience had made him an indispensable officer to the Guardians. The Tribunal, however, dismissed the appeal, but agreed that Mr. Sugden should not be called up for service for two months in order that the Guardians might make arrangements to fill his position.

### SAND-BAGGED HOSPITAL STATUES.

So long ago as June 1916 (THE HOSPITAL, June 24, 1916, p. 291) we recorded that the tomb of Rahere in the Church of St. Bartholomew-the-Great, East Smithfield, a monument precious in the eyes of all hospital folk, had been heavily sand-bagged, and we felt thankful that this precaution against the weapons of flying barbarians had so wisely been taken. Our only regret was that the same method of safety could not be found practicable and applied to the whole of the ancient and beautiful church, which has been restored and preserved with such skill and patience. Elaborate efforts are now being taken to render the statue of Thomas Guy secure from air-raids, and we notice that something of the same kind is being carried out in respect of the statue of Captain Coram, outside the gates of the Foundling Hospital. We should have thought that none would have found fault with such simple and temporary safeguards, but, apparently, the work at the great South London hospital offends the æsthetic feelings of a correspondent to *Guy's Hospital Gazette*, who, moreover (in view of the multiplicity of London's statues), considers that a dangerous precedent is being set up, and looks forward with apprehension to the swathing of Nelson's Column or the Monument.

### SHOULD HOSPITAL BUILDINGS BE MADE BOMB-PROOF?

THE Editor of *Guy's Hospital Gazette*, in referring to the precautionary measures in connection with Thomas Guy's statue, quotes the example of the recently-injured Cleopatra's Needle, thus braving the Censor who, if he lets this breach of the regulations pass, may perhaps allow our hospitals, and there are several of them, to glean public sympathy, as well as material and financial damage, from their misfortunes in air-raids. But another question raised is whether some precautionary work may not be applied to the hospital buildings as well as the hospital monuments. Some of our wards, points out the Editor of the *Gazette*, are easily penetrable, and the closing and sand-bagging of wards and rooms nearest the roof would protect the rest of the building. One authority is quoted as expressing the opinion that if a roof is covered with a sufficient depth of sand-bags the same comparative immunity will ensue. We notice that in Venice, for the protection of ancient buildings, mattresses of seaweed are interposed with the sand-bags; this method may be useful in the case of towns near the sea, but the difficulties of transit would render it impracticable as applied to London. However, to protect British hospitals inside or out might cost a great deal of money. It is possible, therefore, that committees of management may consider the possibility of a direct hit sufficiently unlikely, and decide to go on as they are.

### DISAPPEARING DISEASES

OWING to the improved sanitary conditions in the city of Hereford, diseases such as typhus have been eliminated, and typhoid fever has practically disappeared. Other infectious diseases, such as whooping-cough, measles, chicken-pox, and mumps, will perhaps never be eliminated, as they are, to a large extent, not dependent on insanitary conditions. So remarks Dr. J. W. Miller, the medical officer of health, who proceeds to declare that. although vaccines have been prepared against certain of these diseases, and in all probability will eventually be discovered for all of them, it is very doubtful whether the inoculation of such vaccines will be carried out to any considerable extent. Dr. Miller has grounds for making this assertion, owing to the small number of children who are now being vaccinated against smallpox. Parents of the present day are afraid of the slight degree of discomfort in connection with vaccination, and only a small proportion are willing to have their children's teeth treated at the clinic on account of the slight pain or discomfort that may ensue. There is still a good deal of ignorance in regard to infectious disease, and Dr. Miller suggests that children should not be allowed to commence school until they are five years old, on account of the risk of exposure to infection.

### T.N.T. POISONING.

SINCE the introduction of tri-nitro-toluol as an explosive, cases of poisoning resulting from the absorption of this chemical have occurred, and in 1915 an investigation was commenced into the mode in which this poisoning took place. The inquiry was undertaken by the Applied Physiology Department of the Medical Research Committee of the National Health Insurance Department, in co-operation with the Medical Inspectors of Factories, Dr. Moore conducting the investigation. The report of this inquiry has recently been issued. The T.N.T. can be absorbed through the skin and it can also enter the body through the mouth, but by whichever route it enters the results are the same. The first effect is damage to the red corpuscles of the blood, so that their power of carrying oxygen from the lungs to the tissues is greatly impaired, and thus a condition of anæmia is brought about. However, if the further entry of the poison into the body be prevented, perfect recovery is still possible, especially if an abundance of fresh air is provided. The T.N.T., later, has a destructive action on the liver, and moreover it tends to destroy the organs which form the blood, but the main harmful effect is its action on the blood itself. From the very first recognition of symptoms due to tri-nitro-toluol regulations were drawn up to prevent poisoning, and these have been amended from time to time; as an indication of the value of these regulations the number of cases of poisoning has steadily diminished. It is to be hoped that the work of the investigators will diminish still more the chances of poisoning by this explosive.

### BUSY DENTISTS.

DENTISTS appear to be the busiest people in the land. At the request of the Board of Control the Monyhull Colony Committee of the Birmingham Board of Guardians considered the question of appointing a dentist at the Colony, and invited seven gentlemen in the locality to become candidates for the position. Five of those approached were unable to consider the matter at all, and the remaining two asked for a very high fee. The committee finds that a large number of dentists in the city are on active service, with the result that those remaining are exceedingly busy and unable to undertake any fresh appointments. The Board of Control has been informed of these facts, and the committee has postponed the matter until the end of the war. Meanwhile the medical officer will continue his present practice of dealing with any urgent dental work. The committee, by the way, has received an application from the medical officer for a gratuity in respect of extra duties. Deeming it inadvisable to continue to award gratuities, the committee suggests that the salary should be increased from £150 to £200 per annum, with an allowance of £20 for the services of a dispenser.

### A GROWING COTTAGE HOSPITAL.

THE cottage hospitals of Wales are not excluded from the generosity which is helping the big institutions through their period of growth. In fact, the generosity proceeds from the same source—namely, the magnates and trade organisations of the coalfield. For instance, the Porth Cottage Hospital has received three gifts of one thousand guineas in the past year, whereby three beds have been endowed in the new ward which is expected to be opened shortly. This ward will consist of some thirteen beds, so it is clear that Porth will possess before long a larger institution than a cottage hospital.

### WOMEN TUBERCULOSIS OFFICERS.

THE first women doctors acting as tuberculosis officers under the Staffordshire, Wolverhampton, and Dudley Joint Tuberculosis Committee, lately sent in their resignations because their application for an increase of salary to £500 a year was refused. The committee, which had offered an increase to £450, then decided that a sub-committee should invite the applicants to meet them, and discuss the committee's proposal. If the offer, on reflection, is not accepted, the committee decided to advertise for two chief tuberculosis officers at £500 a year each, and for two assistants at £350. It was contended that in Birmingham, with one exception, no tuberculosis officer was receiving more than £400 a year. Without prejudging the claim of the applicants, it would be interesting to know what special experience and training they have had. So far, tuberculosis, too little studied in any case, has not been specially studied by women as a rule.

### TEHIDY SANATORIUM.

THE fund to establish Tehidy Sanatorium, in Cornwall, is making satisfactory progress, and now that the £10,000 authorised by the county meeting has been promised and in part subsidised, a new effort is being made to raise a further sum to provide timber and other necessaries, and to improve the estate by the purchase of fifty-six acres, known as Park Land. When complete the sanatorium will form the county war memorial, and it is possible that a regular farm colony may be formed.

### "MAY I BRAG?"

HOW many doctors or nurses in the North of England, more especially perhaps in Yorkshire, remember having this question put to them, not by a special appeal secretary or an allotment-holder, but by a convalescing patient at meal-times? The meaning in the real old vernacular is simply "May I have a second helping?"—so that here, as always, the folk speech has the best of it as regards brevity and force. Skeat states that the etymology of the verb "brag" is unknown; the middle-English form was "braggen," which meant to sound loudly, or vaunt. Perhaps a more possible derivation is from the root shown in the Anglo-Saxon *brec*, the Spanish *bragas*, and the Latin *bracae*, which all mean "breeches." At all events a pleasant strain upon these articles of clothing is implied certainly enough in "May I brag?" Another interesting dialect verb is *teem* (=to pour out). It must be the infinitive of which is now extant only the dire *cliché* "teeming," and perhaps the Scotch "toom" (=poured out, thus empty). Some of our readers may have heard both phrases this Christmas and New Year.

### HELP NEEDED AT AIR-RAID SHELTERS.

THERE is still a great need in London for further assistance being rendered by qualified women at the various raid shelters. Although some considerable time has elapsed since they were opened, in some cases, and those more especially in the poorer parts of the Metropolis, no capable women have yet appeared to take charge of the unfortunate persons flocking to the shelter. This was particularly noticeable at Bermondsey Town Hall on the night of the last raid. The place was packed with women and children of the very poor, who came there in the scantiest of garments and unprepared for any emergency. The Mayor some time ago received a promise from the local branch of the British Red Cross Society that two qualified nurses would be sent to the Town Hall on the occasion of a raid, but they failed to attend. The consequence was that there was no woman present capable of attending to a woman who fainted, and was unconscious for a considerable time, and a search of the building failed to find any of the ordinary and commonplace stimulants used in cases of this kind. Such a lack of unpreparedness is greatly to be deplored, especially where 300 to 400 women and children are congregated, and where the ventilation is not sufficient for such huge assemblages. With a view to getting into more direct touch with the Red Cross work in the borough, the Mayor has decided to form a local branch of the society. Hitherto it has been affiliated with the Camberwell branch, but it is evident that if something is to be done, and that quickly, to remove the lack of supervision at present prevalent in the borough, the Bermondsey section of the British Red Cross Society must be directly in touch with the Mayor and his colleagues, so that they may know that capable nurses and qualified women are to be had to take charge of the shelters.

### WOOD FIRES.

The Government announcement that ten million young trees are awaiting planting out, and that unless they are got into the ground during the coming planting season they will die, suggests once more the importance from more than one point of view of timber as fuel. The price and the supply of coal are already regulated, and it is tolerably certain that should the winter be severe and prolonged many a household will find the Government allowance insufficient. A larger use of wood fires in districts where faggots are easily obtainable at a comparatively cheap rate would not only relieve the pressure upon the coal supply—a pressure which must necessarily increase as time goes on—but would obviously conduce to the purifying of the atmosphere. The frequency and density of London fogs have been much reduced by the more general adoption of smoke-consuming apparatus, but the clear aromatic smoke from wood fires has few fog-producing qualities; moreover its volume is much smaller. A house in which logs are habitually burned soon becomes impregnated with the "odour sweet," if not of the "cedar log and sandal-wood" of the *Iliad*, at all events of the oak log and the beech block. Some modern fireplaces, no doubt, require a little adaptation for the comfortable burning of wood, but that is not invariably the case. In London and most large towns, the cost of carriage is against any extensive use of wood fuel, but every coal-burning chimney the less is a help, small though it may be, to the public health, while to the sensuous delight of a wood fire most of us are susceptible.

### VOLUNTARY AGENCIES IN LAMBETH.

The health work of voluntary agencies seems to be much appreciated in Lambeth, where the maternity and child-welfare schemes are carried out through the medium of various voluntary organisations. All local agencies concerned were co-ordinated by a scheme prepared by Dr. Priestley in April 1916, and it is declared that the work carried out by the voluntary agencies is proving satisfactory and that much good is consequently being done throughout the borough, thereby relieving the Council of the necessity of carrying out the whole of this work itself. The voluntary agencies are properly officered with well-qualified medical officers and health visitors or trained nurses; and reports are submitted from time to time to Dr. Priestley, thereby linking up directly this voluntary work with the official work of the Council dealing with maternity and infant-welfare. Lambeth, of course, is exceptionally fortunate in this respect, there being so many organisations in the borough, besides St. Thomas's Hospital, the General Lying-in Hospital, and the Clapham Maternity Hospital, all of which are linked up in the scheme. The Council, too, has the great advantage of its municipal milk depôt, which has proved of great service in infant-welfare. Since the inauguration of the scheme a permanent committee has been appointed in commemoration of Baby Week, and the Mayor of Lambeth is the chairman. The Council now proposes to make grants to some of the agencies associated with this comprehensive scheme, it being pointed out that owing to the war and other conditions voluntary subscriptions are difficult to obtain, and that unless financial help is forthcoming from the Council it may be necessary for some of the voluntary agencies to cease work or to curtail their activities considerably. It may be added that grants to voluntary agencies are made also by the Borough Councils of Islington, Fulham, Hammersmith, and Chelsea.

### JOHANNESBURG BABIES.

Johannesburg babies are to be taken under the beneficent surveillance of the Municipal Council, which is asking the Provincial Administration to give it the powers necessary for the establishment of a Babies' Clinic. Before coming to this decision the Municipal Council had a report prepared of the work done in this direction in England. The special committee which inquired into the subject could not recommend giving a grant to a voluntary organisation, but thought that the municipality itself should establish a clinic. The Local Government Ordinance, however, does not give general powers to local authorities for the establishment of municipal institutions, and so the Council is asking for these new powers. It is noticeable that in matters affecting health most of the municipalities in South Africa and Australia are adopting methods which have already been employed in this country.

### THE THIRD LONDON "GAZETTE."

The January number of the *Gazette* of the 3rd London General Hospital contains names which seem new to us, and, since changes must occur, it is a hopeful sign. The recent departure of Corporal Ward Muir will be a great loss; but a not dissimilar task has been found for him in propaganda, both at home and at the Front. One associates the recognition of special aptitudes with anything rather than war or military service, and therefore it is cheering to find that he has found this fresh extension of usefulness. Miss Nightingale is still among the versifiers; and a new cover design by Lance-Corporal J. H. Dowd is a pleasant invitation to the varied contents of the number.

